# Mechanical Properties and Durability of Textile Reinforced Concrete (TRC)—A Review

**DOI:** 10.3390/polym15183826

**Published:** 2023-09-19

**Authors:** Chao Wu, Yang Pan, Libo Yan

**Affiliations:** 1 Department of Civil and Environmental Engineering, Imperial College London, South Kensington Campus, London SW7 2AZ, UK; 2 School of Transportation Science and Engineering, Beihang University, 37 Xueyuan Road, Beijing 100191, China; panyang@buaa.edu.cn; 3 Centre for Light and Environmentally-Friendly Structures, Fraunhofer Wilhelm-Klauditz-Institut WKI, Riedenkamp 3, 38108 Braunschweig, Germany; 4 Department of Organic and Wood-Based Construction Materials, Technische Universität Braunschweig, Hopfengarten 20, 38102 Braunschweig, Germany

**Keywords:** textile reinforced concrete (TRC), fibre reinforced polymers (FRP) mesh, bond behaviour, mechanical properties, durability

## Abstract

Textile reinforced concrete (TRC) is an innovative structure type of reinforced concrete in which the conventional steel reinforcement is replaced with fibre textile materials. The thin, cost-effective and lightweight nature enable TRC to be used to create different types of structural components for architectural and civil engineering applications. This paper presents a review of recent developments of TRC. In this review, firstly, the concept and the composition of TRC are discussed. Next, interfacial bond behaviour between fibre textile (dry and/saturated with polymer) and concrete was analysed considering the effects of polymer saturation, geometry and additives in polymer of the textile. Then, the mechanical properties (including static and dynamic properties) of TRC were reviewed. For static properties, the mechanical properties including compression, tension, flexural, shear and bond properties are discussed. For dynamic properties, the impact, seismic and cyclic properties were investigated. Furthermore, the durability of TRC under different environmental conditions, i.e., temperature/fire, humidity and wet–dry cycles, freeze–thaw, chemical and fatigue were discussed. Finally, typical engineering applications of TRC were presented. The research gaps which need to be addressed in the future for TRC research were identified as well. This review aims to present the recent advancement of TRC and inspire future research of this advanced material.

## 1. Introduction

### 1.1. TRC Concept and Advantages

**Figure 1:**
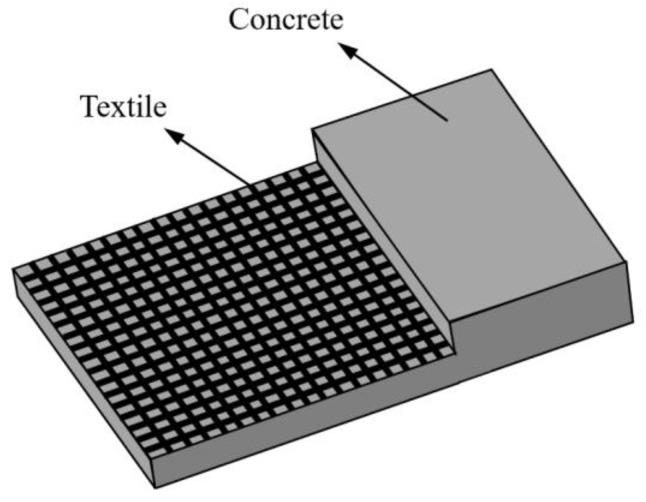
Step-wise cut through the TRC cross-section.

Textile reinforced concrete or TRC is defined as a material which consists of textiles, as internal reinforcement, made of long woven, knitted or even unwoven fibre rovings in at least two directions, embedded in an inorganic fine-grained binder (typically—but not necessarily—cementitious) [1]. A step-wise cut through the TRC cross-section is shown in Figure 1. TRC can be used for manufacturing thin structural parts with complex shapes [1]. Compared with the traditional steel reinforced concrete, TRC has no risk of electrochemical corrosion of reinforcing materials in the environment with salt ions [1,2].

### 1.2. FRP Textile for TRC

Fibre reinforced polymer (FRP) is a composite material composed of fibre reinforcement embedded in the polymer matrix [3]. FRP composites have many advantages, such as high strength/weight ratio, light weight, corrosion resistance, good durability, flexibility in shape and tailorability [3]. FRP has been used for strengthening/retrofitting existing civil engineering structures since the 1980s [3,4]. Afterwards, various FRP materials and structures have been extensively used globally, including: (1) pultruded FRP structural members (e.g., FRP beams, FRP columns, FRP tube confined concrete and FRP decks) and full FRP structures (e.g., FRP bridges and FRP cooling towers), (2) filament winding structural members (e.g., chimney liner, oil pipelines) and (3) FRP sandwich panels [3]. It was estimated that the glass FRP (GFRP) market will grow to $82.1 billion by 2027 at a Compound Annual Growth Rate of 6.4% [5].

The physical and mechanical properties of various fibres of FRP are given in Table 1. Natural fibre has a lower density than other fibres. Carbon fibre has the highest tensile strength, followed by glass fibre, basalt fibre, aramid fibre and boron fibre. In addition, natural fibre has the lowest tensile strength. The comparison in service temperature indicates that the service temperatures of glass fibre, basalt fibre, boron fibre, aramid fibre and carbon fibre are higher than polyethylene fibre and natural fibre. 

**Figure 2:**
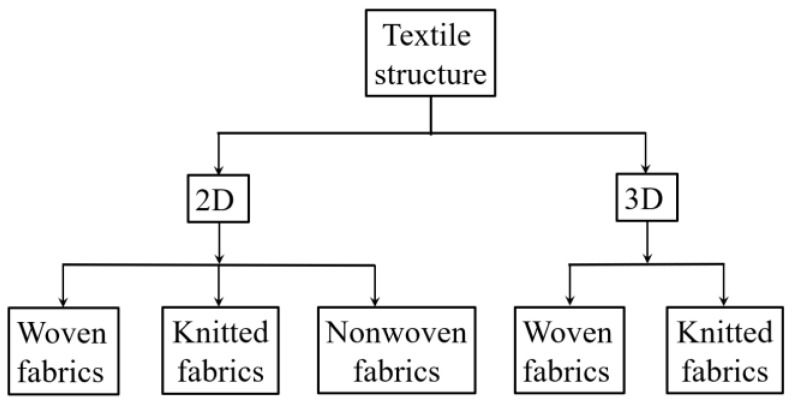
Typical textile structures.

**Figure 3:**
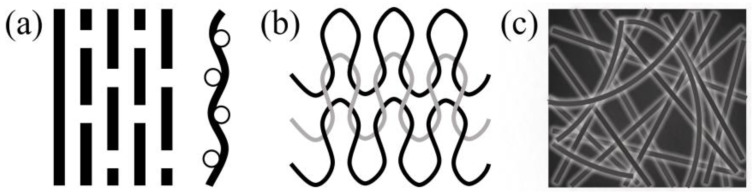
Two-dimensional textile structure produced by different method of fabric formation (**a**) woven fabrics, (**b**) knitted fabrics and (**c**) nonwoven fabrics.

The typical textile structures are shown in Figure 2 [1]. Two-dimensional textile has the fibres or yarns arranging in the same plane [1]. They can be mainly categorized into woven fabrics, knitted fabrics and nonwoven fabrics (Figure 3) [1,24].

**Figure 4:**
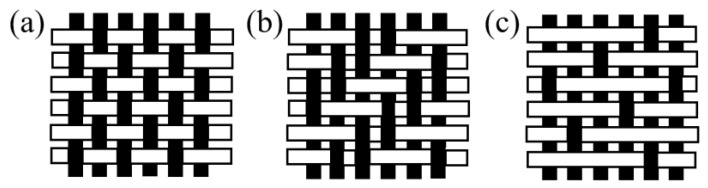
Different weaving patterns: (**a**) plain weave, (**b**) twill weave and (**c**) satin weave.

Woven fabrics are composed of two sets of yarns, warp and weft, that are formed by weaving, which is the interlacing of these sets of yarns [25]. The yarns are held in place by the inter-yarn friction [24]. Three basic weaving patterns used are plain, twill and satin weave (Figure 4), and the weaving pattern affects the properties such as stability of displacement of the fabric [1,25]. For instance, the plain weave has the highest stability of displacement because it has the shortest floating (how often a thread is crossing threads of other system on a certain length) [1,25]. The decrease in the stability of displacement may cause the threads to skip from their position in later process steps [1,25]. Typically, fabrics with an open and grid structure can be called Leno fabrics [1]. These fabrics have two twisted warp yarns around a weft yarn [1]. The advantages of the leno weave include: (1) high dimensional stability, i.e., when it is used as the concrete reinforcement, the mesh size could change from 5 mm × 5 mm to 20 mm × 20 mm, (2) realization of open, grid-like structures [1]. Weaving is the most common form of interlacing, and woven fabrics still occupy a dominant position among all kinds of textile fabrics, because of the established tradition [1,24]. Woven fabrics are typically made of alkaline-resistant (AR)-glass, carbon or basalt, or, most-recent, plant-based natural fibres such as flax [1]. Flat wovens of full or half cross weave are mostly used to improve the tensile properties [26,27], shear capacity [28], flexural behaviour [26,29,30] and toughness [30,31,32] of woven fabrics reinforced concrete.

Knitting is another form of interlacing where the thread in one set interlocks with the loops of neighbouring thread by looping [24]. Structure and the properties of the knitted fabrics could be adjusted for different applications, i.e., from highly stretchable to almost inextensible, with fixed sizes; from thin, open structure to dense; from smoothly surfaced to nonplanar arrangements with textured or embossed surfaces [1]. Concrete reinforced with open grid warp knitted fabrics with polymer coating has been used in the hypar shell structure of a building in Germany, with carbon filament yarns in the warp and weft directions [33,34]. Three layers of textile fabrics were added in the fine-grain concrete, with a total thickness of 15 mm [33,34]. However, compared with woven fabrics, knitted fabrics did not develop so well [35]. This is because they have two drawbacks: lower mechanical properties caused by the bending of the fibres and low volume fraction in concrete due to the specific geometry of knitted stitches, characterised by areas without yarns [35]. 

The nonwoven fabric is defined as a manufactured sheet or web structures bonded together by entangling fibres or filaments, by various mechanical, thermal and/or chemical processes [36]. It has an isotropic nature, large porosity and it would provide a homogeneous surface [1]. With open structures, it can provide excellent anchorage and bond behaviour in cement reinforcement and it can increase the volume content of fibre in composites without side effects [37].

**Figure 5:**
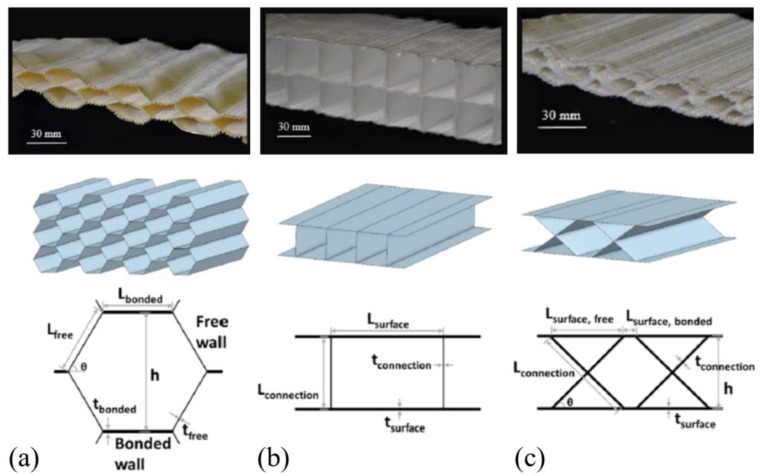
(**a**) Fabrics with profiled surfaces and hexagonal cavities, (**b**) Fabrics with flat surfaces and rectangular cavities, (**c**) Fabrics with flat surfaces and X-shaped connections [48].

Three-dimensional textile has three directions in yarn architecture and/or textile architecture, regardless of whether it is made in a “one-step-process” or “multiple-step-process” [25,38]. In addition, it could be also categorized into woven fabrics and knitted fabrics [1]. Weaving technology can also be used for the manufacture of 3D textiles [24]. Three-dimensional textile structures are typically made by processing two-dimensional woven fabrics into a three-dimensional structured shape, e.g., by co-curing and joining [39,40,41,42], stitching [43,44,45] or z-pinning [46,47]. Compared with 2D textile fabrics, 3D textile structure has improved mechanical properties, such as high energy absorption, low crack propagation, good impact behaviour and high tensile strength [24,48]. Three major geometries of 3D woven textiles include fabrics with profiled surfaces and hexagonal cavities, fabrics with flat surfaces and rectangular cavities and fabrics with flat surfaces and X-shaped connections (Figure 5) [48].

**Figure 6:**
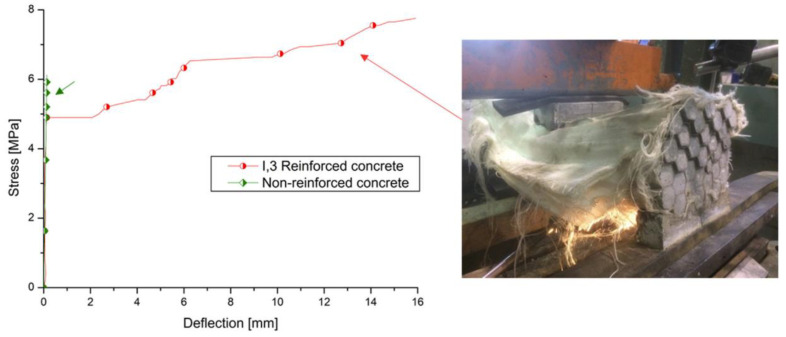
Stress–deflection curve and failure pattern of the 3D high tenacity (HT)-polyester fabric reinforced the concrete under the three point bending test [48].

Geerinck et al. [48] investigated the stress–deflection curve of 3D high tenacity (HT)-polyester fabric reinforced concrete under three-point bending test. Compared with non-reinforced concrete, the 3D fabric reinforced beams showed a very different failure pattern (Figure 6) with the ability to sustain loading at a much higher deflection capacity [48]. The ductility of the 3D fabric reinforced beams was improved significantly due to the restriction of the crack propagation because of the existence of the internal fabric [48]. 

The main advantages of using 3D textile as reinforcing material of concrete are that it offers better impact resistance than the 2D textile counterpart (because 3D textile structure has the crimpless structure compared with 2D textile structure), high formability due to their drape characteristics, high complexity shapes [35,49,50]. However, it has been rarely investigated and used because the development of 3D knitted fabrics is still at the laboratory stage, because the mechanical properties are relatively low. For instance, the tensile breaking strength of 3D knitted polyethylene (PE) fabrics is about 300 N. The tensile strength test is used to pinpoint the warp and weft strength of the fabric [49]. In addition, the specific properties of 3D textiles are hard to predict due to the complexity of knitted fabrics and uneven behaviour of the final composite, and the pretension of their performance before its impregnation with resin [35]. Furthermore, the production speed of 3D textile structure is also relatively slow [51].

### 1.3. Inorganic Matrix for TRC

**Figure 7:**
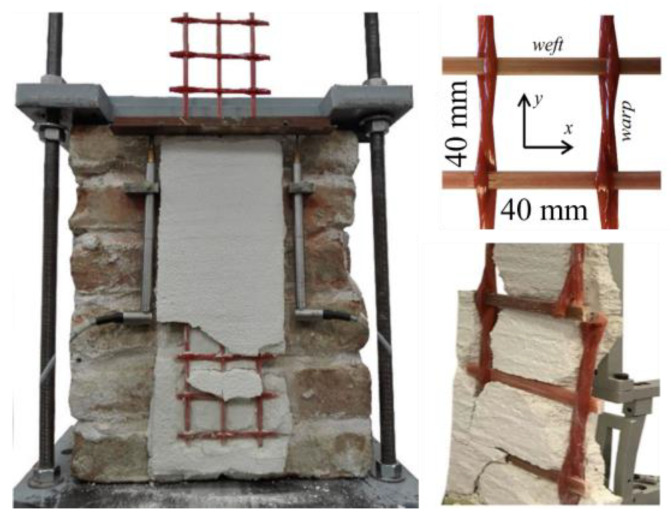
Structure of CRM.

It needs to be clarified that textile (or only fibre) reinforced inorganic matrix composites are referred to with different names in this research, including fibre-reinforced cementitious matrix (FRCM), textile reinforced mortar (TRM) and textile reinforced concrete (TRC) [1]. In addition, there is a strengthening system comprising FRP grids and inorganic matrices named composite reinforced mortar (CRM) [52,53]. FRP grids have a mesh-like fibrous structure that allows the mortar to protrude through the grid’s openings (Figure 7) [52].

Fine-grained concrete with a maximum grain size (<2 mm) is mostly used for TRC. Depending on the distance between the yarns of the mesh, the maximum grain size of highly flowable consistencies concrete is 0.6 mm [1,25]. The water/binder (w/b) ratio is usually between 0.3 and 0.4, and the binder content is usually 40–50% by volume [1]. Compositions of some frequently used fine grain concrete are listed in Table 2 [1]. 

Acceptance criteria, design and standard guidelines on inorganic–matrix composites have been investigated in several design guidelines including “Guide to Design and Construction of Externally Bonded Fabric-Reinforced Cementitious Matrix and Steel-Reinforced Grout Systems for Repair and Strengthening of Concrete Structures” (ACI 549.4R-20) [54], “Guide to Design and Construction of Externally Bonded Fabric-Reinforced Cementitious Matrix (FRCM) and Steel-Reinforced Grout (SRG) Systems for Repair and Strengthening Masonry Structures” (ACI 549.6R-20) [55], “Linea Guida per la identificazione, la qualificazione ed il controllo di accettazione dei sistemi a rete preformata in materiali compositi fibrorinforzati a matrice polimerica da utilizzarsi per il consolidamento strutturale di costruzioni esistenti con la tecnica dell’intonaco armato CRM” (Composite Reinforced Mortar) [56], “Externally-bonded composite systems with inorganic matrix for strengthening of concrete and masonry structures” (EAD 340275-00-0104) [57], “Acceptance criteria for masonry and concrete strengthening using fabric-reinforced cementitious matrix (FRCM) and steel reinforced grout (SRG) composite systems” (AC434) [58] and “National Research Council. 2020. Guide for the design and construction of externally bonded fibre reinforced inorganic matrix systems for strengthening existing structures” (CNR-DT 215/2018) [59]. 

High workability is normally required for concrete of TRC, so self-compacting fine-grain concrete has been investigated in recent years [60,61,62]. Self-compacting fine-grain concrete with average compressive strength about 31.8 MPa was adopted for TRC, and it was expected to be used in masonry structures [61]. Self-compacting concrete (the maximum grain size of sand is 1.2 mm) was used to prepare TRC for high workability in Ref. [60]. In order to adequately penetrate the textile reinforcement grid structure, so as to contribute to the quality of the bond between the matrix and the reinforcement, self-compacting concrete was used to prepare TRC for new building external walls [63].

Fine-grained ultra-high performance concrete (UHPC) with textile reinforcement enables concrete elements with a minimum thickness [64]. UHPC thin panels reinforced with carbon fabric pre-impregnated with epoxy resin were tested to investigate flexure [65]. However, to produce high-quality UHPC requires very precise proportion control of raw materials, temperature control and optimization of the mixer, which is extremely difficult in the realization process [66,67]. It is necessary to investigate the effect of the textile reinforcement on the construction of TRC with UHPC [66,68,69,70].

Textile-reinforced recycled aggregate concrete (RAC) has not been extensively investigated [71,72]. However, according to the latest review of mechanical performance of RAC in 2022, polypropylene (PP) fibre-, glass fibre (GF)- and basalt fibre (BF)-reinforced RAC have been investigated widely. The compressive strength, splitting tensile strength, durability of fibre reinforced RAC has been discussed in the past 10 years [71]. However, unlike normal aggregate concrete, the residual properties of RAC are lower (i.e., RAC containing 100% of RCA displays a significant loss in relative compressive strength, because the microstructure of RAC consists of weaker interfacial transition zones owing to the adhered cement mortar to the surface of RCA), fibre reinforcement can effectively prevent and retard the microcracks via fibre crack-bridging in RAC, and thereby results in the improvement of residual mechanical properties (e.g., RAC reinforced with 1% and 2% volume fractions of PP fibre was found to produce about 15.6% and 35.7% greater compressive strength than that of RAC without fibres) [71,73].

**Figure 8:**
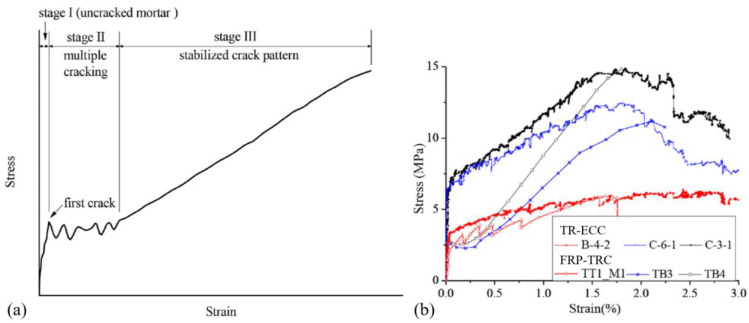
(**a**) Typical axial tensile stress–strain curve of TRM, (**b**) Comparison of TR-ECC and TRC under direct tensile load [76].

Engineered cementitious composites (ECC) can also be used for making TRM. ECC has been developed with a strain capacity up to 8% based on the micro fracture mechanics. The ductility of ECC is achieved with microcracks (the width of the microcrack was less than 60 μm) [74,75]. When ECC is applied in textile reinforced ECC (TR-ECC) (Figure 8), it has supreme tensile strain capacity and exhibits strain hardening in tension due to the inherent short fibres’ bridging effect [76,77]. Tensile strain of TR-ECC (over 3%) is almost twice of that of FRP-TRC (about 1.7%) [76,77].

The main challenge of the matrix of TRC or TRM is reducing the binder content, specifically the Portland cement, to be ecological and economical, because cement production contributes at least 5–8% of global carbon dioxide emission, and it requires high temperature (1450 °C) decomposition of the raw materials to generate reactive calcium silicate and aluminate phases [1,78]. 

### 1.4. Scientometric Analysis of the Literature Items of Textile Reinforced Concrete

Bibliometrics is a statistical method that could analysis the key areas of research with software such as VOSviewer. The web of Science (WOS) online database includes important research, and the search results of “textile reinforced concrete” from WOS online database could be exported with VOSviewer.

The global literature about the textile reinforced concrete was scanned in all databases of WOS. The information of the research that meet the requirements includes title, author, affiliation, keywords, year of publication, journal and abstract, and all of this information was exported. The retrieval date was 26 August 2023. VOSviewer (version 1.6.19) was used to analyse the co-occurrence, bibliographic coupling and themes. 

**Figure 9:**
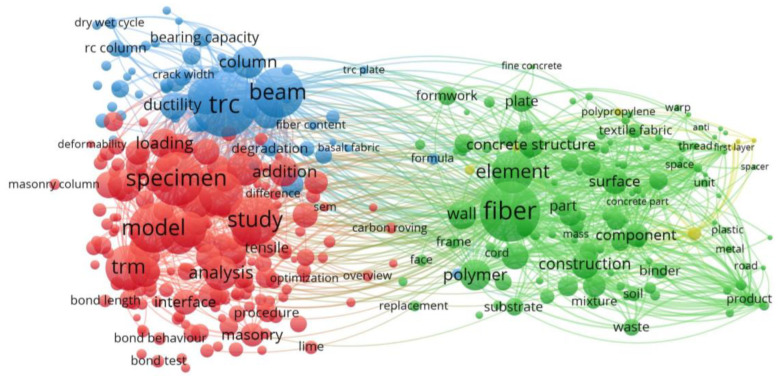
Bibliometric analysis of the topic in publications of textile reinforced concrete.

A total of 2844 publications on the topic of “textile reinforced concrete” were identified. Bibliometric analysis of the topic in these publications is shown in Figure 9. The size of nodes indicates the frequency of occurrence. The curves between the nodes represent their co-occurrence in the same publication. It is found that the published literature is focused mainly on the topics of interface bond between FRP textile and concrete matrix, mechanical behaviours and durability of TRC.

### 1.5. Structure of the Review

This review will be structured in five main sections, as follows:

Section 2 focus on the discussion and analysis of interface bond between FRP textile and concrete matrix of TRC. In this part, the effect of FRP textile (i.e., with or without resin and geometry of the textile), and effects of additives in textile for improved interface bond are introduced firstly. 

Section 3 discusses the mechanical behaviours of TRC, including static properties (i.e., compressive properties, tensile properties, shear properties, flexural properties and bond properties) and dynamic properties (i.e., impact properties, seismic performance and performance under cyclic force). 

Section 4 contains durability of TRC, including effects of temperature and fire, humidity, freeze–thaw cycles, chemical condition, creep and fatigue. 

Section 5 outlines the practical applications of TRC in non-structural elements, structural elements and structural rehabilitation. 

Section 6 is the outlook including durability and nano research, design code, new materials and new structure and new applications, i.e., 3D printing, airport, tunnels and special traffic.

## 2. Interfacial Bond between Textile and Concrete Matrix

The textile–matrix interfacial bond strongly influences the mechanical properties of TRC [1]. A strong bond usually leads to the fracture of the yarns, providing a high-strength composite with low ductility; whereas a weak bond leads to the pullout of yarns, providing a lower strength composite with higher ductility [1].

### 2.1. Effect of Textile on the Bond

#### 2.1.1. With or without Resin

**Figure 10:**
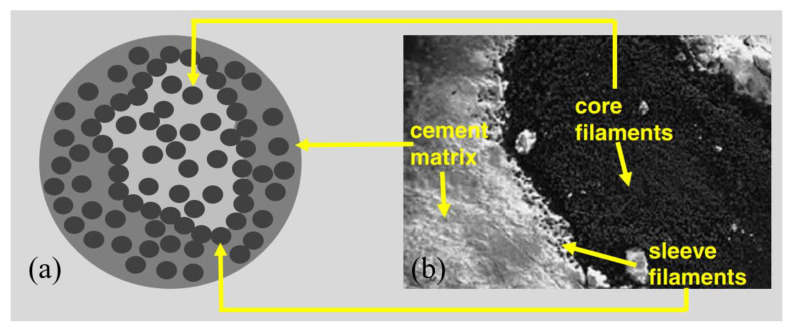
Cross-section of a bundle embedded in cement matrix: (**a**) schematic view and (**b**) actual carbon bundle in cement matrix.

The epoxy-impregnated fabrics exhibit better bond resistance in the concrete, compared with the fabrics without resin. This is due to the fact that if the spaces between the fibre filaments are fullfilled, better stress transfer among the fibre filaments can be achieved to provide higher load bearing capacities [1,79,80,81,82,83]. The mechanism was analysed and proved with scanning electron microscope (SEM) observations. An SEM image (Figure 10) of a cross-section of a carbon bundle in cement matrix was used to investigated the cement grain penetration into the bundle [79]. As can be seen in Figure 10, the cement paste cannot fully penetrate into the inner filaments. The fibre bundle was composed of multiple filaments with inner gaps less than 5 μm. However, the size of the cement grain was typically 5~70 μm, which was much larger than the gap among the filaments, so that gap among the filaments could not be fully filled with the cement grains [79]. To avoid alkali degradation, the GFRP textile with epoxy-impregnated bundles is recommended; in this case, the acidic glass filaments could be kept away from the alkaline cement matrix to avoid the chemical corrosion during ageing [1].

#### 2.1.2. Geometry of the Textile (Mesh or Grid)

**Figure 11:**
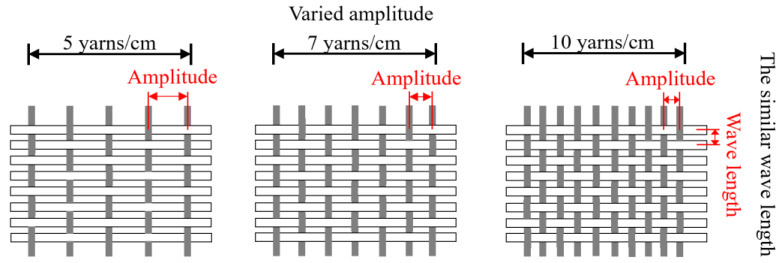
Woven fabric with different amplitudes and similar wave length.

The use of FRP mesh reinforcement has been explored over the past two decades due to its flexibility to be fabricated into complex shapes [84,85]. The nature of the interactions between the concrete and textile mesh (or grid) is more complex than the interactions between the concrete and sheet textile [86,87]. Textile mesh differs mainly in the junction points of the connected yarns. Appropriately embedding more junction joints of the fabrics in the concrete could improve the bond strength between the textile mesh and the concrete [88]. Commonly used mesh sizes are 5 mm × 5 mm to 25 mm × 25 mm [1,24,84]. The yarns in woven fabric are crimped, and this shape has an increased effect on the bond behaviour (pullout tests were used to investigate the bond between the fabric and the cement matrix, straight single yarn was also tested for comparison, the bond strength of a yarn in a fabric was about seven times of the bond strength of a straight yarn) [89]. However, the multifilament geometry of knitted fabric has a negative effect on the bond behaviour (bond strength of fabric < the bond strength of a straight yarn), due to the penetration of the matrix in the complex fabrics being limited [89]. The effect of the wave amplitude of crimp geometry of the individual yarn in woven fabric on the bond behaviour with matrix has also been investigated [89,90]. Pullout behaviour of two crimped yarns with different amplitudes and similar wave length has been tested (Figure 11). The specimens with the larger amplitude have higher pullout load, indicating that larger wave amplitude had contribution to the bonding behaviour, because the larger wave amplitude had a better anchoring effect [89,90]. 

### 2.2. Effect of Additives in Textile on the Bond

**Figure 12:**
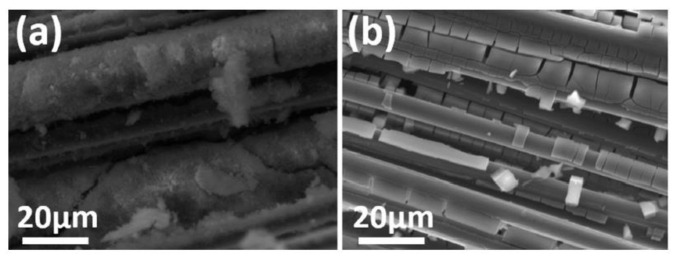
Detailed ESEM images (**a**) uniform microsilica coating, (**b**) non-uniform nanosilica coating [95].

Filling the spaces (or gaps) among the filaments could improve the bond behaviour between the textile fabric and concrete matrix, because the stress transfer among the filaments becomes better [1,79,80,81,82,83]. Filling nano particles (with small particle size) into the voids among the filaments could improve the durability by preventing the deposition of the hydrates between the filaments [91,92,93]. The bond strength of AR (alkaline-resistant) glass yarns in cement matrix under accelerated aging was investigated. A drastic improvement of the bond behaviour was achieved by adding the nanoparticles of microsilica with particle size of 50 nm in AR glass yarns [94]. For carbon yarns, filling the spaces among carbon multifilament yarns with microsilica, nanosilica or epoxy resin was investigated to improve the bond behaviour based on the pullout test. The filling with microsilica particles was found to be the superior option, compared to epoxy coated specimen [95]. This is because the active microsilica boosted the Ca^2+^ in the space of the filaments to generate uniform calcium-silicate-hydrate (C-S-H) products, whereas the nanosilica coating was not uniform (Figure 12) [95]. The continuous polymer coating creates efficient bonding between the individual fibres, whereas bonding between the yarns and the concrete matrix is insufficient, due to the absence of C-S-H formation or penetration between them [95].

The effects of textile properties, such as with/without resin, geometry and additives on the bond between textile and concrete matrix, have been investigated widely, and the bonding mechanism has also been explored. However, the research on the concrete properties, e.g., strength and aggregation, on the bond is limited, and thus is encouraged in future studies. 

## 3. Mechanical Behaviours of TRC

### 3.1. Static Behaviour

#### 3.1.1. Compressive Properties

**Figure 13:**
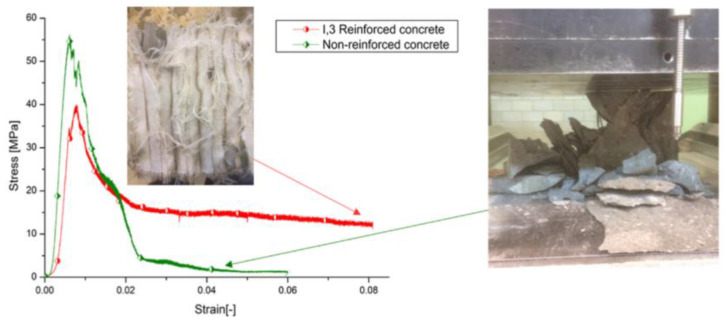
Comparison of the failure mode of fabric reinforced the concrete and non-reinforced concrete under compression [48].

The compressive strength of the reported compressive strength of glass textile TRC, basalt textile TRC and carbon textile TRC mainly ranges from 30 MPa to 111.46 MPa [1,48,84,96,97,98,99]. For example, the compressive strength of the TRC shell cross-section with a thickness of 20 mm and 6 layers of carbon textiles was 68 MPa [99]. Geerinck et al. [48] investigated the stress–deflection curve (Figure 13) of the 3D fabric reinforced concrete under compression test, compared with the specimens of non-reinforced concrete. The fabrics were produced with high-tenacity polyester yarns (110 tex), para-aramid yarns (110 tex) or glass yarns (136 tex), at full width of the loom (150 cm) [48]. The compressive strength (edge-wise compression tests) of the non-reinforced specimens (≈56 MPa) was higher than the one of 3D fabric reinforced concrete (≈40 MPa). The non-reinforced concrete failed at the first crack; however, fabric reinforced concrete presented ductile behaviour with a large deformation capacity. The research on the compressive strength of TRC is limited. The standard methods for TRC compressive strength testing need to be explored and proposed, and the mechanism of the compressive failue needs to be further investigated [48,84,96,97,98,99].

#### 3.1.2. Tensile Properties

**Figure 14:**
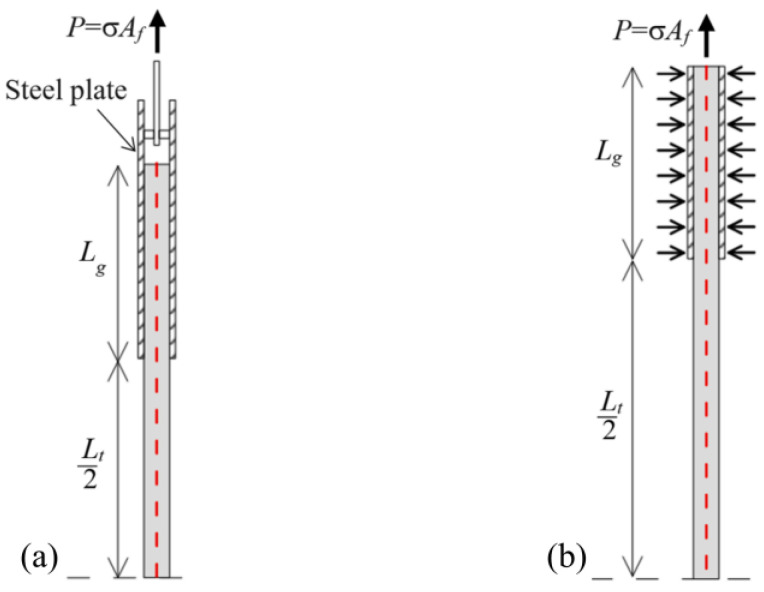
(**a**) Side view of a clevis-grip test; (**b**) side view of a clamping-grip test [102].

Different tensile test set-ups are proposed in the literature to analyse the tensile properties of TRC composites [100,101,102]. According to the US acceptance criteria AC434 [58], tensile test of TRC should be performed with the clevis-grip set-up, where steel plates are attached to the end of the specimen and then connected to the testing machine with a clevis joint (Figure 14a). According to the Italian acceptance criteria [56] and European assessment document EAD 340275-00-0104 [57], tensile test of TRC should be performed using the clamping-grip set-up, where the specimen ends are gripped by the machine wedges applying sufficient pressure to prevent matrix–fibre slippage within the gripped length (see Figure 14b). In both these test set-ups, the composite axial stress σ is computed as the ratio between the load P applied to the specimen and the textile cross-sectional area A_f_, while the specimen axial strain ε should be measured with an extensometer mounted to the central portion of the specimen [102].

**Figure 15:**
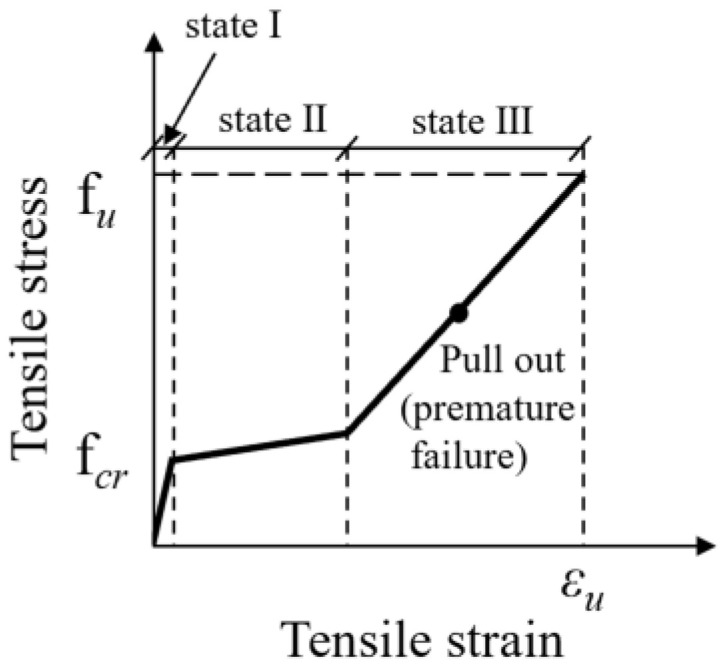
Typical tensile stress–strain relationship of TRC.

Direct tensile testing based on rectangular specimens with two displacement transducers (LVDTs) was proposed to investigate the tensile properties of TRC, the displacement rate was 1 mm/min was selected [103]. It has been reported that tensile strength of TRC shell reinforced by AR-glass fabric or carbon textiles was mainly 8.05 MPa to 49 MPa [77,84,98,104]. Higher tensile strength could be obtained by increasing the layers of carbon textiles or compacting fabric–matrix interface by filling the bundle spaces with epoxy first and then coating the filled fabric with silica particles [77,104]. Tensile properties of TRC of four types of fabrics (E-glass, AR glass, basalt, aramid and carbon) were tested, respectively, and the failure pattern of these TRC is shown in Figure 15. The failure pattern of these TRC has been classified into three states: State Ⅰ (uncracked concrete), State Ⅱ (multiple cracks form) and State Ⅲ (sample reaches the ultimate load, brittle tensile failure is observed) [103,105,106]. Tensile behaviour of TRC at the microscopic level was also discussed. The tensile response was considered at three levels as the filament, crack bridge and direct tensile tests. The tensile and bond behaviours could be determined based on the stress distributions of TRC [84,107]. Tensile properties of TRC were also investigated in Ref. [108]. After the formation of the first crack, additional cracks also initiate. As applied load increased, more cracks formed until no more cracks could develop due to the fibres not being able to transfer the load back into the matrix [108]. After the textile were pulled out, the specimens were eventually destroyed. The tensile energy absorption capability was attributed to multiple dissipation mechanisms including formation of crack surfaces, interfacial debonding, pull-out and failure at the mechanical anchorage points [108].

**Figure 16:**
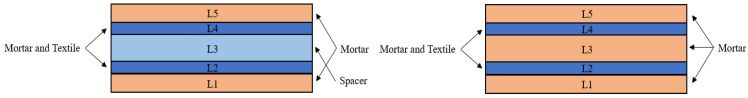
Schematic comparison between the cut 3D and 2D AR-glass fibre textile TRC.

**Figure 17:**
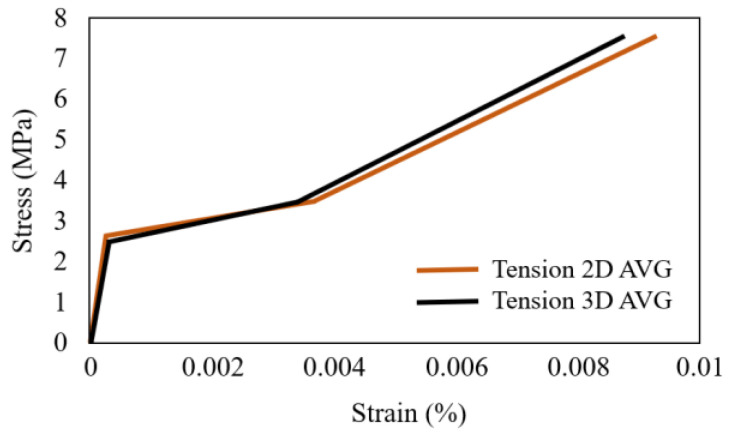
Tensile averaged curves of 3D and 2D AR-glass fibre textile TRC.

Tensile properties of 3D and 2D AR-glass fibre textiles reinforced cementitious matrix have been compared [109]. The tensile tests were performed on Instron 5885 loading machine with a rate of 2 mm/min. Properties of 3D and 2D AR-glass fibre textiles are shown in Table 3. Schematic comparison between the 3D and 2D AR-glass fibre textile TRC is shown in Figure 16. Tensile averaged curves of the 3D and 2D AR-glass TRC are shown in Figure 17. Three-dimensional and 2D AR-glass fibre textile TRC exhibited a similar tensile behaviour in the precracked stage. E-modulus of 3D and 2D AR-glass fibre textile TRC were 10,736.27 MPa and 10,972.63 MPa, respectively [109]. The matrix cracking stress of 3D and 2D fibre textile TRC were 2.28 MPa and 2.44 MPa, respectively, and matrix cracking strain were, respectively, 2.13 × 10^−4^ and 2.22 × 10^−4^. This similar behaviour of 3D and 2D AR-glass fibre textile TRC is expected, because the composite mechanical response is governed by matrix in the elastic stage [109]. The contribution of the spacer in the 3D AR-glass TRC’s compared to the 2D AR-glass TRC’s was investigated through comparing the post-cracking E-modulus (respectively, 752.49 MPa and 722.08 MPa) [109]. The test results showed that the spacer of 3D AR-glass fibre textile TRC did not improve the resistance against pull-out of the fibres for the tensile experiments [109]. The tensile properties of 3D and 2D AR-glass fibre textile TRC are similar [109].

#### 3.1.3. Shear Properties

Research on shear strength of TRC is scarce. Indeed, the shear properties of TRC and the shear failure mechanism need to be clarified in future [84,96,110]. TRC shear design for new constructions remains an important subject of current research [111]. Longitudinal splitting and delamination occurred in the specimens under the shear load due to the good bond characteristics between the textile and the matrix [111]. Shear capacity of TRC slabs was discussed, where carbon-textiles with epoxy impregnation were used to reinforce the cementitious matric. The test results indicated that planar textile grids and pre-formed C-profiles increased shear capacity despite small slab thickness (120 mm) through their distributed reinforcing function aligned to member direction [111]. Shear tests were also conducted to investigate the shear properties of the RC beams repaired with TRC, through four-point-bending test with load control at a speed of 600 N/s. The global behaviour of the RC beams repaired with TRC is quite similar to those repaired by CFRP, e.g., the ultimate strength gains obtained with the TRC reinforcement can reach 38% [112]. U-wrapping CFRP strip and TRC were, respectively, used to strengthen the shear capacity of short-span concrete beams reinforced with GFRP bars [113]. The test results showed that the shear strength of specimens strengthened with TRC and CFRP increased by about 33.7% and 12.1%, respectively [113]. In addition, the number of textile layers in TRC slightly affected the shear capacity of the tested specimens due to the crushing failure of the concrete struts [113].

#### 3.1.4. Flexural Properties

**Figure 18:**
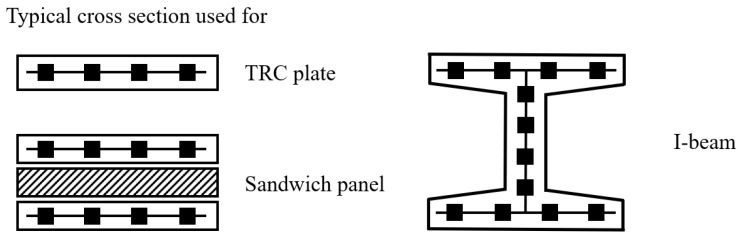
Typical cross-section of TRC elements.

**Figure 19:**
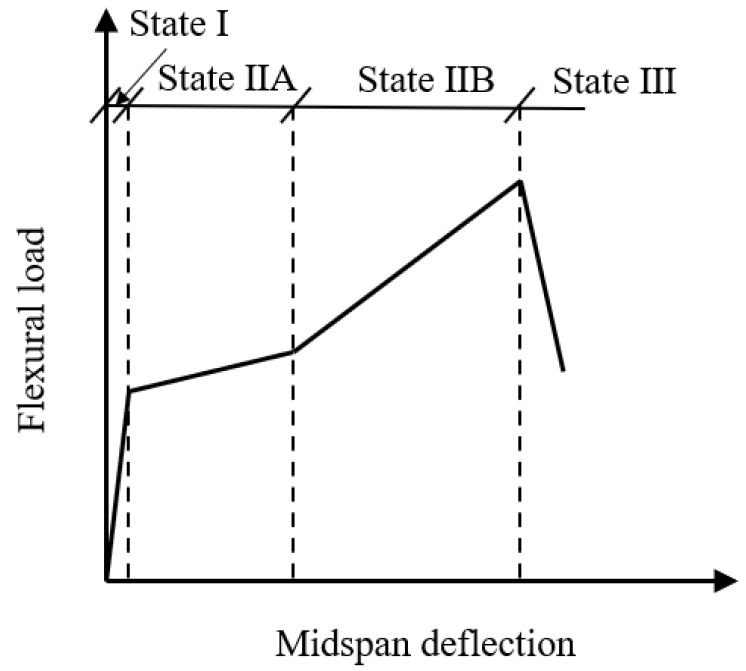
Flexural load versus mid-span deflection curve with indicated stages.

The flexural properties of TRC have been widely investigated, because most TRC elements are used for tension members (such as shells and facades), and failures were observed in bending [84]. The flexural strength of TRC was tested under three-point and four-point loadings, and TRC specimens could sustain a smaller flexural strength because they are thin, compared with normal concrete panels or beams [84]. It has been reported that flexural load of TRC reinforced with carbon fabric is still less than 10 kN, so that the failure of TRC under bending load should be monitored appropriately in the future [1,84,114,115]. The bending capacities of TRC plates [1,114,115], sandwich panels [116,117] and I-beams [118] were investigated. Typical cross-sections of TRC elements are shown in Figure 18. Flexural behaviour of TRC plate reinforced by carbon fabric was investigated by four-point bending tests [115]. Four states were typically defined for the load–displacement response, as illustrated in Figure 19: State I (uncracked concrete), State IIA (crack formation), State IIB (crack stabilization) and State III (failure) [1,114,115]. The bending capacity of TRC could be improved by pre-stressing textiles. Test results showed that the first-crack load capacity of the specimens with pre-stressing textile was increased by 85%, compared with the non-prestressed specimens [119].

The flexural properties of 3D and 2D AR-glass textiles reinforced cementitious matrix are also compared. Four point bending test was performed with a span of 350 mm, and the distance between the loading pins was 100 mm. The flexural tests were performed on Instron 5885 loading machine with a rate of 2 mm/min [109].

**Figure 20:**
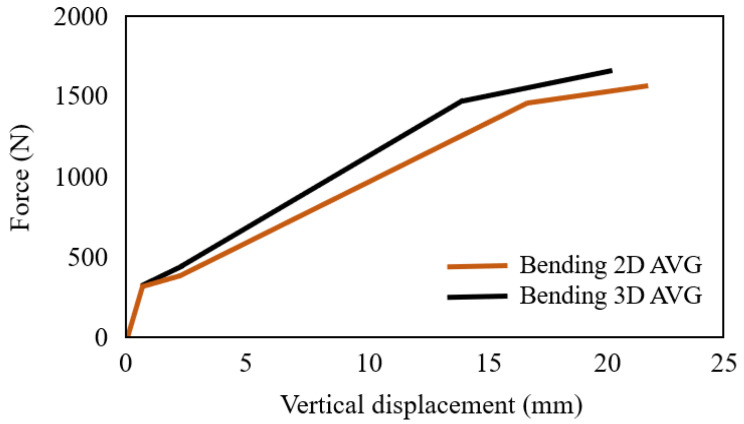
Flexural averaged curves of the 3D and 2D AR-glass TRC.

Bending averaged curves of the 3D and 2D AR-glass textile TRC are shown in Figure 20. The spacer material of 3D AR-glass textile TRC did not have a noticeable impact on the elastic stage. However, in the post-cracking stage, compared to 2D AR-glass textile TRC, the stiffness of 3D AR-glass textile (91.51 N/mm) is 25% higher than that of 2D AR-glass textile TRC (73.07 N/mm). The increase in stiffness of the 3D AR-glass textile TRC may be due to the three-dimensional fibre fabrics optimizing the change in stress distribution over the thickness, as the fibre volume fraction of 3D and 2D AR-glass textile TRC are the same [109]. 

#### 3.1.5. Bond Properties

The interfacial bonding properties between TRC and masonry substrate were analysed using a single shear test, and the most common failure mode was tensile rupture of textile [77]. The ultimate load and bond strength were the largest when the bond length exceeded the effective bond length that was related to the textile types [77]. With the same TRC matrix, the effective bond length of carbon textile, coated-carbon textile and basalt textile were 100 mm, 150 mm and possibly less than 50 mm, respectively [77]. The effective bond length was also investigated in [120], and the effective bond length was considered closely related to the stiffness and geometric properties of TRC [120].

The stress transfer mechanism between the matrix and textile was investigated experimentally using direct and indirect shear tests and then described analytically with various models [121,122,123]. Due to the high strength of the fibres, failure of externally bonded fibre-reinforced composites was usually due to debonding [121]. To understand the bond behaviour of fibre-reinforced composites, a rigid-trilinear cohesive material law was proposed to describe the frictional stresses at the interface of debonding [121]. The analytical solution of composite–substrate joints with different bonded lengths and with free and fixed far end was provided. A mesoscale three-dimensional non-linear finite element approach was employed to investigate the bond behaviour of FRCM composites. Matrix and fibre bundles were explicitly modeled to take into account both matrix–fibre interaction and matrix–textile interlocking [122]. Results of single-lap direct-shear tests of stitch-bonded carbon FRC–Mmasonry joints were compared with the results of the numerical approach. Stress concentrations at longitudinal–transversal fibre bundles caused the cracking of the matrix, which led to the specimen failure, and similar results were also captured by the numerical model [122]. The results of the peak load obtained from the numerical model and that estimated by an analytical procedure assuming no interaction between transversal and longitudinal bundles showed that the presence of stitch-bonded joints did not improve the FRCM-masonry joint load carrying capacity; however, further research is needed to confirm these results with different bonded lengths and other FRCM composites [122]. Two simplified numerical models are proposed to simulate the tensile and shear bond behaviour of FRCM composites [123]. Both models took advantage of truss and non-linear spring elements to simulate the material components and the interface, and the proposed approach deduced the global mechanical response in terms of stress–strain or stress–slip relations [123]. From the comparisons of the numerical results with the experimental benchmarks from the literature, it emerged that the interface constitutive laws play a key role in the global response of the specimens [123]. The numerical model was able to reproduce the observed experimental tensile and shear bond behaviours: both the tensile- and shear-bond 1D model were able to reproduce the experimental trend well, especially in the fibre–mortar combination [123].

Bond properties between TRC-confined seawater sand concrete and BFRP bars under chloride corrosion environment were discussed. BFRP bars were buried in the corner of the C40 seawater sand concrete specimen (35 mm away from the edge of the specimen, refer to the Standard for Test Methods of Concrete Structures (GB/T50152-2012) [124,125]). Before being reinforced with TRC, the surface was roughened to expose the coarse aggregate with a roughening depth of 2–3 mm, after that, a layer of carbon-fibre woven mesh was wrapped circumferentially along the stress direction of carbon fibre, and then a layer of fine-grained concrete with a thickness of 2–3 mm was applied in turn [125]. The research results showed that TRC confinement could change the failure mode of specimens, and with the increase in layers of textiles, the failure mode changed from brittle split failure to ductile pull-out failure [125].

### 3.2. Dynamic Properties

#### 3.2.1. Impact Properties

Few studies have been reported on the properties of TRC under impact load [1]. This might be interpreted by the fact that research and application of TRC were mostly in architectural elements and structural strengthening and repairing [1]. More research is expected in this area, considering the advantages of TRC. The impact behaviour of the TRC reinforced with glass fabrics and polyethylene (PE) fabrics was investigated in Ref. [126]. The pultrusion method was used to produce the TRC [126]. The impact properties were investigated with the set up of three-point bending with a drop weight system. The results showed that the fabric in the composite was quite effective on the impact load carrying capacity and deflections at mid-span [126]. The impact fatigue behaviour of GFRP mesh-reinforced ECC was investigated for the application of runway pavement [127]. Under the impact load of the design aircraft pressure, after being impacted with 30,000 times, the test results showed: (1) the bottom of GFRP mesh reinforced ECC generated several microcracks without failure, (2) the bottom of ECC without reinforcement generated more cracks and (3) the slabs of concrete quickly broke after 10 impacts [127]. 

TRC with 3D fabric was studied under impact. Three-dimensional fabrics consisted of two sets of independent two-dimensional knitted fabrics, which connected with a third set of yarns along the thickness direction [1]. Two loading arrangements of vertical and horizontal to fabric layers were carried out, respectively [1]. In the horizontal arrangement, the two faces of the 3D fabric were located at the top and bottom of the specimen relative to the drop direction of the hammer, where the spacer yarns were passing through the thickness from top to bottom [1]. In the vertical arrangement, the two faces of the 3D fabric were located at the sides of the specimen relative to the drop of the hammer, and the spacer yarns were passing through the width of the composite between the two sides of the composite [1]. The toughness of the vertical system was much greater than that of the horizontal system, providing much better energy absorption of the vertically tested composite. However, the horizontal system exhibited greater maximum stress as compared to the vertical system [1].

#### 3.2.2. Seismic Performance

The seismic performance of PVA textile TRC permanent formworks reinforced full-scale reinforced concrete (RC) circular columns were investigated, considering the superior tensile and ductility properties of TRC [128]. The test setup constituted a column fixed to the laboratory floor, a steel loading beam for constant axial load, MTS hydraulic actuator for cyclic loading and linear variable differential transducers (LVDTs) for displacement measurements [128]. The specimens were tested under a constant axial load and an incremental reverse cyclic lateral load. The lateral load capacities and energy dissipations of the columns constructed with TRC formwork were about 1.2 times and 1.8 times of the RC columns reinforced with conventional temporary formworks [128]. Under the seismic load, the columns constructed with TRC formwork provided stable crack propagation and distribution by parallel multiple cracks; however, the concrete cover of RC columns without TRC spalled [128]. Seismic performance of TRC strengthened RC columns was studied in Ref. [129]. The results showed that TRC could delay the stiffness degradation of the strengthened columns with a large axial compression ratio and a small shear span [129]. Under the reciprocating load, the accumulated energy dissipation of TRC-strengthened columns (37 kN/m) was much more than that of the columns that were not strengthened (22 kN/m), which indicated that TRC could significantly improve the seismic performance of the specimens, the textiles consisted carbon fibre bundles and glass fibre bundles and the mesh size was 10 mm × 10 mm [130]. The effect of textile layers on the seismic behaviour of TRC-strengthened RC columns was investigated with the numerical method [131]. Within the range of 1 to 3 layers of textile grid layout, the peak load and displacement ductility of TRC strengthened columns increased with the increase in the number of layers, although the increased range was limited. For example, the peak load of the specimens with 1 to 3 layers of textile grid layout were 119.92 kN, 121.80 kN and 124.08 kN, respectively. The displacement ductility of the specimens with 1 to 3 layers of textile grid layout were 36.21 mm, 42.52 mm and 45.59 mm, respectively. With the increase in the number of textile grid layers, the energy dissipation rate of the specimen also increased slightly, e.g., the energy dissipation capacity of the specimens with 1 to 3 layers of textile grid layout were 33,085.75 kNmm, 46,881.53 kNmm and 48,895.22 kNmm, respectively. In addition, the number of textile grid layers had no obvious influence on the yield load, e.g., the yield load of the specimens with 1 to 3 layers of textile grid layout were 107.77 kN, 110.75 kN and 111.73 kN, respectively.

#### 3.2.3. Performance under Cyclic Force

The properties of glass textile TRC and carbon textile TRC structures under cyclic loading were investigated in the last decade [132,133,134]. The experimental equipment used in this work comprised a Zwick machine to apply tensile and cyclic loads, optical fibre sensors and DIC. All tests including the monotonic test, the cyclic front-loading test and the post-cyclic residual test were controlled at a speed of 0.1 mm/min, while the cyclic test was controlled at a velocity of 5 mm/min [133]. Cyclic behaviour of TRC under different loading modes (cyclic tensile loading in the precracking stage, cyclic tensile loading in the crack propagation stage) was investigated [134]. The global behaviour of the TRC subjected to cyclic loading in precracking stage showed an elastic behaviour without cracking [133]. Regarding the global behaviour of the TRC subjected to cyclic loading in the crack propagation stage, the stiffness of the TRC decreased as the number of cycles increased [133]. The new cracks caused an instantaneous decrease in the global stiffness of the TRC [133]. Cyclic loading in the crack propagation stage led to a decrease in the stiffness of precracking stage of TRC [133]. The cyclic behaviour of RC columns strengthened with TRC was also discussed. Specimens reinforced with TRC had a better performance compared with the specimens reinforced with FRP sheet of the same reinforcement ratio [132]. Effect of adding PVA fibres on the tensile cyclic behaviour of TRC (with alkali-resistant glass fabrics) was investigated, and the results showed that the presence of PVA fibres in the mortar mixture helped in controlling the cracking of TRC [134].

Many experimental studies have been conducted to investigate mechanical properties of TRC. In terms of static properties, the characteristics and mechanisms of tensile and flexural properties of TRC were widely discussed, and the shear and bond properties were also extensively studied based on different kind of textile and concrete matrix. However, there is a lack of standard test method and mechanism analysis on compressive properties of TRC. In terms of dynamic performance, impact performance of TRC, the seismic performance of TRC reinforced old structures and the properties of TRC under tensile cyclic loads have been investigated. In the future, it is also necessary to pay attention to the dynamic performance of 3D textiles-TRC exposed to different loading conditions.

## 4. Durability of TRC

### 4.1. Temperature and Fire

The properties of 2D and 3D textile reinforced concrete at high temperature have not been extensively researched in the literature [1,135]. Based on the existing literature, the thermal response of TRC materials could be assumed to be similar to the ordinary Portland cement concrete, and the fabrics in concrete have a less significant influence on their thermal properties [1,114,136,137].

Under the temperature of about 400 °C, the mechanical properties of 2D textile reinforced concrete decreased rapidly [1,135,138]. A thermo-mechanical machine (TM20kN-1200C) was used to investigate the behaviour of 2D textile reinforced concrete under high-temperature loadings. The test procedure consisted of homogeneously heating the specimen to a desired temperature level and applying a monotone uniaxial tensile loading on the specimen until its rupture [139]. Sulfo-aluminate cementitious matrix reinforced with five layers of alkali-resistant-grid glass textile and chopped strand mat (GRI) was heated from 20 °C to 400 °C under tensile load; when temperature increased from 20 °C to 300 °C, the initial tangential stiffness of the GRI decreased, and the ultimate stress of the GRI reduced from 7 MPa (20 °C) to 1.8 MPa (400 °C) [139]. When the temperature reached 200 °C, the tensile strain of GRI was about 0.9%, and when the temperature reached 400 °C, the strain was only about 0.1% [139]. All ruptured specimens were characterized by a macrocrack that was almost perpendicular to the direction of the tensile force. When the temperature was between 200 °C and 400 °C, the macrocrack was located at the submerged portion of the specimen in the furnace, and a rupture was quickly followed by an opening of the lips of this macrocrack [139]. 

**Figure 21:**
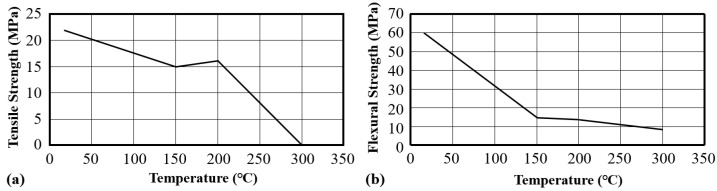
(**a**) Tensile strength of 3DT-RCC at high temperatures. (**b**) Flexural strength of 3DT-RCC at high temperatures.

The properties of 3D textile reinforced mortar exposed to high temperature were also investigated [138]. The thickness of the 3D textile reinforced mortar specimens was 8 mm, the top of the 3D fabric was covered with thick linen fabric and the bottom was covered with rubber; the inside of 3D fabric consisted of polyethylene terephthalate (PET) yarns, and calcium aluminate cement binder was confined inside [138]. Three-dimensional textile reinforced mortar specimens were exposed to 150 °C, 200 °C and 300 °C for two hours, then tensile and flexural tests were performed [139]. Melting of some parts of the yarns led to a decrease in the tensile strength of 3DT-RCC at the 150 °C, and 3D textile reinforced concrete lost its bearing capacity at about 300 °C (Figure 21) [139].

It has been recommended that two methods can be used to improve the fire resistance of TRC: (1) increasing the thickness of the concrete cover, (2) using carbon fibres as reinforcement since carbon fibre has high temperature tolerance up to 1500 °C. However, these two methods also increase the cost and/or self-weight [1,114]. Attention should be paid in future research to improve the fire resistance of the matrix (i.e., develop and characterize the fire-resistant geopolymer matrix), and investigate the thermal and mechanical properties of it under the load in compression, tension and shear [1].

### 4.2. Humidity and Wet–Dry Cycles

Research on the effect of humidity on behaviour of TRC is limited [1,140,141]. The tensile properties of alumina cement-based mortar reinforced with uncoated CFRP mesh (TRC-ALC) under a high temperature and humidity environment (50 °C and 95% relative humidity) were investigated. TRC coupons were stored at a constant temperature of 50 °C and relative humidity of 95% in a chamber for 60 days, then the direct tensile test was performed one day later [141]. The stiffness of TRC-ALC under the temperature of 50 °C and relative humidity of 95% decreased obviously, compared with the specimens exposed at room temperature and humidity (COM). The tensile strength of TRC-ALC (2.7 MPa) was about 0.5 MPa lower than that of COM (3.2 MPa), and the tensile strain of TRC-ALC (1.1%) was about two times that of COM (0.5%). During the tensile test, cracks appeared at the middle part of the TRC coupons, then more cracks appeared and continued to widen, until failure [141]. AR-glass TRC, basalt TRC and carbon TRC were kept in water at 50 °C for 10 days, then the flexural characteristics of these TRC specimens were tested (flexural tests were executed in a deformation-controlled testing machine at a strain rate of 1.8 mm/min (ASTM C947-03) [142]. The test results showed that, for the specimens with the textiles coated with styrene butadiene copolymer, the flexural stress of AR-glass TRC, basalt TRC and carbon TRC did not changed obviously (the flexural stress of AR-glass TRC before and after aging were 57.0 MPa, 55.1 MPa; the flexural stress of basalt TRC before and after aging were 314.7 MPa and 304.0 MPa; the flexural stress of carbon TRC before and after aging were 93.4 MPa and 97.4 MPa) [143]. However, it should be pointed out that for the carbon TRC specimens, whose textiles were coated with both styrene butadiene copolymer and micro silica, the flexural stress increased by about 16.5% after curing for 10 days in water of 50 °C (the flexural stress of carbon TRC coated with both styrene butadiene copolymer and micro silica before and after aging were 164.7 MPa and 191.8 MPa) [143].

The durability of fibre-reinforced cementitious matrix and composite-reinforced mortars subjected to wet–dry cycles were discussed with single-lap direct shear tests [144]. The fibre-reinforced cementitious matrix was composed of carbon fibres, polybenzene terephthalate isoxazole (PBO) and alkali-resistant (AR) glass fibres embedded in the cementitious matrix [144]. Composite-reinforced mortars were made of AR glass composite grid and natural hydraulic lime. Specimens of fibre-reinforced cementitious matrix and composite-reinforced mortars were exposed to 50 wet–dry cycles prior to testing [144]. The test results showed that the average peak stress of these specimens remained almost unaltered [144]. It should be noted that the continuation of the hydration of inorganic matrix composites with glass fibre textiles could cause the penetration of hydration products within the voids among the filaments, which may lead to a reduction in the matrix–fibre bond properties, so more studies are needed to identify the dominant effect among these two competing factors [145].

### 4.3. Freeze–Thaw Cycles

The mechanical properties of glass TRC (quartzite aggregates with a maximum aggregate size of 600 μm were used for the concrete) under freeze–thaw cycles have been investigated [146]. The specimens were subjected to freezing–thawing cycles according to ASTM C 666 [147]. The temperature varied between +4 °C and −18 °C (the cooling and heating rate were all 11 °C/h, and the temperature at +4 °C and −18 °C was kept for 30 min). Cycle times of 25, 50, 75, 100, 150 and 500 were considered [146]. Both uncracked and precracked specimens were tested [146]. The results showed that when the cycle number exceeded 100 times, the degradation of the TRC (uncracked specimens) occurred, and cycles affected the ultimate tensile strength of the specimens, for uncracked specimens, when 500 cycles were performed, about 80% of the initial maximum tensile stress was reached; however, the tensile stress of precracked specimens’ variation ranged between 0.8 and 1.2 of its initial maximum tensile stress without a clear trend [146]. The mechanism of this phenomenon was discussed, the tensile stress was reduced by the damage due to the thermal cycles; however, the self-healing and late hydration of the matrix can increase the strength of the specimens due to the cracks in precracked specimens, which facilitates the penetration of water [146].

Effect of chloride freeze–thaw cycles on the bending behaviour of hybrid TRC (carbon bundles was used in the warp direction and glass bundles was used in the weft direction) was investigated [148]. The method for the chloride freeze–thaw cycles was as follows: the lowest and highest temperatures in the centre of the specimens were (−18 ± 2) °C and (5 ± 2) °C, respectively, each freeze–thaw cycle lasted 3 h and the 5% NaCl solution was used in this test. After 90 chloride freeze–thaw cycles, some material of the fine grained concrete peeled off from the surface and edges of the specimens [148]. After 50, 70 and 90 freeze–thaw cycles, the ultimate bending load noticeably decreased (the ultimate bending load after 50, 70 and 90 freeze–thaw cycles were 1.15 kN, 1.16 kN and 0.75 kN, respectively) [148]. The main reason was that the chloride freeze–thaw cycles caused the freeze expansion damage to the concrete, and lead to the broken of pores in concrete and cracking on the surface of the specimens [148].

It was reported that the substitution of Portland cement by the fly ash or blast furnace slag would reduce the frost resistance of TRC, because the freeze–thaw durability of concrete has a close relationship with its air void parameters (air void parameters of the recommendations are normally 1%~6% total air and spacing factor ≤ 0.20 mm), and the addition of microparticles of fly ash or blast furnace slag in the matrix can contribute to obtain the required air parameters [1,149]. So, ECC (which contains a large content of fly ash) showed an excellent frost resistance. After 300 freeze–thaw cycles, there was only a slight reduction in ultimate flexural stress and ductility (before and after the freeze–thaw test, the flexural stresses of ECC were 11.44 MPa and 9.7 MPa, respectively, and the flexural deflections were 5.23 mm and 4.91 mm, respectively) [150]. 

### 4.4. Chemical Conditions

When TRC is used in salt condition, salt ions may corrode the fibres and the concrete matrix [1]. Conventional glass could be corroded by alkaline environments [1]. AR-glass, which contains 16–20% zirconium dioxide by mass, showed significantly enhanced resistance in highly alkaline environments [1]. In addition, organic polymer sizes applied to filament surfaces during the production of the glass yarns can delay such corrosion significantly [1]. Similarly, basalt fibre exhibited lower alkali resistance in comparison to AR-glass fibre, but adding zirconia improved the chemical resistance of basalt fibre reinforced TRC [1]. Carbon fibre is well known for its high chemical resistance; no degradation of properties could be observed in the usual chemical environments [1].

The dry/wet cycle test method was mainly used to simulate chloride ion corrosion in TRC [151,152]. The TRC specimens were intermittently immersed in chloride solution, and chloride ions naturally penetrate the concrete [151]. A 5% NaCl solution was used, and one cycle involved 12 h of chloride immersion and 12 h of natural drying in air. Test results showed that, TRC could inhibit the intrusion of chloride ions due to the good self-compaction and anti-permeability characteristics of fine-grained concrete [153]. However, the strength of the concrete still would be decreased in the salt condition, because salt ions Cl^−^ and SO_4_^2−^ would react with Ca^2+^ and Al^3+^ in the cementitious material or the hydration products of the cementitious material and generate the hydrates breaking the microstructure of the specimens, and the concrete cracked [154]. If the cementitious material for TRC contains less content of Ca^2+^ and Al^3+^, the durability of the specimens showed better resistance in the salt condition [154].

### 4.5. Fatigue Behaviour

To refine and enhance the service performance of TRC composites, fatigue behaviours of TRC need to be investigated. Nevertheless, the fatigue study of TRC remains a little-discussed topic [88]. The behaviour of multilayer TRC composites under axial tensile cyclic fatigue was investigated. The tensile fatigue behaviour of TRC configurations with fatigue loads of 60% and 80% of the rupture load over 100 cycles was tested [88]. The experimental results showed that the load level did not affect the degradation mechanisms, but the global deformation increased due to the increase in initial deformation. The size of the mesh (4 mm or 7 mm) has no significant effect on the fatigue performance of TRC. However, the improved performance of dissipative capacity of the composite was observed with the addition of carbon and/or glass rods [88]. High-cycle fatigue performance of TRC was tested. The cyclic area increased with the progressive damaging and the rupture of fibre filaments. Failure eventually occurred due to the reduction in fibre cross-sectional area, which made the TRC could not withstand the applied load [155]. Sandwich beams with TRC faces were loaded 100,000 times with four-point bending [156]. The core of sandwich beams was expanded polystyrene (EPS) with a thickness of 200 mm, and the EPS was covered with a 5 mm thick TRC layer on both sides. In addition, the textile mesh of TRC was made of AR glass [156]. It was concluded that the fatigue behaviour of the sandwich beams was strongly dependent on the fatigue behaviour of TRC: no degradation was observed in the core and the bond between EPS and TRC restricted the widths of the cracks [156].

The durability of TRC has also been widely studied in recent years, i.e., the tensile properties and failure modes of TRC at high temperatures have been investigated, and the concrete matrix with fire resistance deserves more attention to improve the fire resistance of TRC. The durability of TRC under the single environment or coupling action of humidity, freeze–thaw cycle, salt erosion and fatigue loads have also been preliminarily discussed. Long-term durability tests need to be carried out, because compared with the design life of the runway pavement (30 years), bridge and building (50 to 100 years), the current research results of the durability of TRC are very limited. 

## 5. Practical Applications of TRC

The use of TRC can reduce the consumption of concrete by up to 85%, and the construction cost and time could be minimized due to the fact that the members can be prefabricated [1]. The practical applications of TRC include: (1) architectural elements; (2) all TRC structure and structural elements; (3) strengthening of the structures and (4) repairing of the structures [1].

Thinner TRC products were used as architectural elements, which could minimize the costs of transportation and installations. The first thin-walled ventilated facade system was built in Germany [7]. Fydro company produced TRC ventilated façade elements for the projects in Germany and Netherland with the area of 17,500 m^2^ and 700 m^2^, respectively [26]. To obtain a more compact and lightweight panels, a self-supporting TRC sandwich façade was investigated and built in the laboratory hall of RWTH Aachen University [5]. Compared with the conventional one, the TRC sandwich façade decreased the weight by approximately 17% and the primary energy consumption associated with nonrenewable resources by about 45%. The respective savings in global warming potential (expressed as kg CO_2_-equivalent/m^2^) amount was approximately 53% [27]. The standard for testing the external facing panel of the buildings was given by the reference [157], including strength, weathering, cold resistance, bonding test, water ponding test, static and dynamic water pressure resistance, water vapor transmission, air barrier resistance, drainage efficiency and flame spread index and smoke development. As the architectural TRC only has been applied for about 15 years [1], the long term properties of the applications need to be further investigated.

The first TRC bridge was built in Germany. Glass fibre and carbon fibre textiles were used to prevent the corrosion of steel reinforcements and thus reduced the weight of the structure [29]. In 2010, Germany built another TRC pedestrian bridge to replace a severely corroded reinforced concrete bridge. The new bridge is 97 m long [158]. Prefabricated TRC garages was built to replace the traditional precast garages. The thicknesses of the walls and the ceilings were reduced to 40~50 mm and 60 mm, respectively, which decreased the weight from approximately 40 t (if made by conventional RC) to about 15 t and saved the transport costs [5]. In 2014, the platform of a rail station was restored with TRC in Germany, to minimize the influences of the rail traffic. Thin and flexible prefabricated TRC plates were used [1]. Furthermore, a roof of five TRC barrel-vault shells over a bicycle stand was investigated. Under the radial load, finely distributed cracks generated to the edge of the shell before the failure crack appeared, and ultimate strain could be up to 0.85% [1]. A composite pipe consists of an inner polymer pipe and an outer ring of TRC was manufactured, which was used for water supply and sewage disposal. The outer ring of TRC improved the loadbearing capacity of the pipe [1,159,160].

TRC has been used mostly for strengthening existing steel reinforced concrete structures [1]. In 2006, TRC was used to strengthen the steel-reinforced concrete with the shape of hyper-shell against the damage of the wind and snow loads [161]. TRC also was used to reinforce the barrel vaulting of a tax office (a historical monument) in Germany. The original structure had to be protected, with the requirements of a flexible and thin reinforcement for strengthening, so TRC was adopted [161]. In 2009, TRC was also used to strengthen the ceilings of a building in Prague, Czech Republic by the company TORKRET. Considering the limited room heights and increased load, TRC was considered to be the best choice [1,162]. Similarly, a ceiling of a production hall in Germany was reinforced with TRC due to its low self-weight [5,36].

TRC also has been applied for strengthening the unreinforced masonry structures, which could be destroyed seriously under high or moderate intensity earthquakes or high wind loads [1]. In 2008, a historic church of Spain was strengthened by TRC. The church was made of masonry, stonework and timber. Serious cracks caused by the earthquakes were generated on the main vault, and TRC was used to strengthen the extrados of the vault to prevent the further development of the existing cracks and the opening of new cracks [1]. In 2012, TRC was also reported to strength a historic unreinforced masonry chimney in France. As the wind load was the most critical load, the 10 mm thick TRC with a layer of carbon textile weighted 168 g/m^2^ was used to strengthen the chimney [5]. In 2013, the main dome of Molla Celebi Mosque in Turkey was strengthened with TRC, because TRC was compatible with the masonry materials of the dome [1], moreover, it also had excellent resistance to fire [5]. In 2014, some masonry walls of a unique building in Turkey, which was built in 1884, needed to be strengthened against earthquake loads. As the building was a historical structure, traditional strengthening techniques, such as adding RC shear walls, could not be implemented, and in turn TRC was considered [5].

TRC is also suitable for the repair of structural cracks [1]. In order to ensure the purity of the sugar and maintain the use of a sugar silo in Germany, TRC was needed which minimized the difficult transport of the building materials into the silo [5]. TRC was used to restore the sewage system in Germany due to the advantage that TRC could be applied flexible in shape and thinner in thickness. Its finer crack distribution lowered the permeability for liquids, and the dense concrete matrix also limited the penetration of pollutants [5]. TRC was used to repair a weir which was built in 1929. Considering the risk of water ingress, un-reinforced mortars could lead to detachment or failures in adhesion. Thus TRC was used to repair this weir, which guaranteed that no significant cracks were observed after two years even in the cycle area [1,163].

TRC was also chosen to confine the concrete support base of a piece of equipment in an industrial plant in the midwestern United States [54,55]. Because the ambient temperature of the concrete support base was about 82 °C, and the temperature was too high for conventional FRP repair [54,55]. The concrete substrate was grinding to provide a good bonding surface firstly. During the installation, the temperature was at approximately 60 °C, so the surface was wetted constantly to apply the matrix layer, then the mesh was pressed into the initial layer of mortar, at last, a third crew followed with the top mortar layer [54,55]. To provide proper curing, a polymer coating and wet burlap were installed [54,55].

## 6. Conclusions and Future Outlook

This paper presents a state-of-the-art review of TRC in terms of its properties and durability. The concept of TRC was discussed and typical FRP reinforcements and commonly used concrete matrices were presented. Then the bond behaviour between FRP reinforcement and concrete matrix was reviewed considering the effect of polymer saturation of the FRP reinforcement, geometry of FRP reinforcement and additives in polymer of FRP reinforcement. Afterwards, the mechanical properties of TRC were reviewed including static and dynamic properties. For static properties, the compression, tension, flexural, shear and bond properties were discussed. For dynamic properties, the impact, seismic and cyclic fatigue properties were included. Furthermore, the durability of TRC was reviewed including temperature/fire, humidity and wet–dry cycles, freeze–thaw, chemical and fatigue conditions. Finally, some engineering applications of TRC were presented. This review highlighted the desirable advantages of TRC structures which offer a solution to material saving through lightweight construction. However, there are still some research gaps that need to be addressed. The key conclusions and future outlooks are presented below:

(1) The durability and interfacial bond of FRP mesh and concrete need to be further improved. Concrete with more fly ash or blast-furnace slag could be used as the matrix of TRC to improve the corrosion resistance in salt condition and the frost resistance under the freezing and thawing cycles. Nanoparticles could be used to fill in the space of the filaments to improve the bond strength of the aging FRP mesh in the concrete.
(2) Fine grain concrete is an important option to maximize the light weight advantage of TRC. Light weight concrete (i.e., contain more fly ash, silica fume) could be used to reduce the weight of the TRC. Using the fly ash, which is the industrial waste, could also be benefit for the environmental protection.
(3) Natural fibre textile reinforced concrete is promising to further reduce the embodied carbon of TRC for green constructions. Although the tensile strength of the natural fibre is relatively lower compared with synthetic fibres such as carbon, due to the low cost and low carbon footprint, natural fibre FRP mesh may be used to reinforce the concrete as the architectural elements or the structure elements with a requirement of lower design load.
(4) Long-term test needs to be carried out for better understanding on the durability of TRC. For example, the design life of the runway pavement [164] is 30 years, and design life of bridge [165] and building [166] are 50 to 100 years. However, long term (30 years) performance records of TRC are very limited.
(5) Three-dimensional textiles need to be explored to further improve the dynamic properties of TRC. Three-dimensional textiles need more research and applications in the future due to their excellent impact assistance properties. Three-dimensional textiles with various type of fabrics and matrix need to be investigated in different conditions.
(6) Self-healing and self-sensing properties of TRC are important for smart infrastructure development. There are no studies related to fabric self-healing in TRC; however, ECC may be used as the matrix of TRC in harsh conditions due to its advantage of self-healing. Carbon fibre could be considered to provide the self-sensing properties in TRC due to its electrical conductivity.

## Figures and Tables

**Table 1:** Physical and mechanical properties of various fibres.

Fibre Type	Relative Density (g/cm^3^)	Tensile Strength (MPa)	Service Temperature (°C)	Chemical Corrosion	Price/kg (USD)
Glass fibre	2.5–2.6 [6]	2400–3450 [7]	<846~1056 [8]	Be corrosion in alkalinity solutions [9]	2 [10]
Basalt fibre	2.67–2.8 [10,11]	2800–3200 [11,12]			1.5–3 [13]
Boron fibre	2.3–2.6 [14]	2100 [14]	<1000 [15]	Corrosion resistant [15]	0.8–4.8 [16]
Aramid fibre	1.44 [10]	Up to 2700 [7,17]	<500 [15]	Low UV resistance [15]	1–1.5 [13]
Polyethylene fibre	0.92 [17,18,19]	103 [17,19]	<85 [17,19]	Chemical resistance and UV resistance [17,19]	2.35–3 [13]
Carbon fibre	1.4–2.0 [10]	2000–6000 [7,17,20]	<1300 [20]	galvanic corrosion of aluminium and steel in moisture [20]	8–14 [10]
Alfa fibre	0.89 [21]	35 [21]	<210 [22]	bond strength between natural fibres and polymer could be improved by alkali to change the acidity surface of natural fibres. [23]	-
Hemp fibre	1.48 [10]	270–900 [21]	<210 [22]		1.55 [10]
Coir fibre	1.15–1.25 [10]	95–230 [21]	<210 [22]		0.2–0.4 [10]
Kenaf fibre	−1.4 [10]	223–930 [21]	<210 [22]		0.378 [10]
Jute fibre	1.30–1.48 [10]	320–800 [21]	<210 [22]		0.926 [10]
Flax fibre	1.4 [10]	343–2000 [21]	<210 [22]		3.11 [10]
Sisal fibre	1.3–1.4 [10]	363–700 [21]	<210 [22]		0.65 [10]

**Table 2:** Compositions of frequently used fine grain concrete [1].

No.	Type	Composition											
		Cement (Type, kg/m^3^)	Fly Ash (kg/m^3^)	Silica Fume (kg/m^3^)	Silica Slurry (w:s = 1:1) (kg/m^3^)	Binder (c + f + s) (kg/m^3^)	Plasticizer (% by Mass of Binder)	Sand (kg/m^3^)			Water (kg/m^3^)	PVA Fibre (kg/m^3^)	HRWRA (kg/m^3^)
								0–0.125 mm	0.2–0.6 mm	0–1 mm			
1	PZ-0899-01 SFB 532	CEM I 52.5 490	175	35	-	700	1.0–1.5	500	715	-	280	-	-
2	FA-1200-01 SFB 532	CEM I 52.5 210	455	35	-	700	0.9	470	670	-	280	-	-
3	M1 SFB 528	CEM III/B 32.5 539	243	-	53.9	809	2.1–2.2	-	-	1079	242.7	-	-
4	M3 SFB 528	CEM I/B 32.5 R 549	246	-	54.6	822	2.3–2.4	-	-	1092	245.6	-	-
5	M7 SFB 528	CEM I 32.5 R 839	-	-	-	839	2.1–2.2	-	-	1189	279.7	-	-

**Table 3:** Properties of 3D and 2D AR-glass fibre textiles [109].

Type	Material	Grid Size (mm × mm)	Tensile Strength (MPa)	Density (g/m^2^)	Spacer Distance (mm)
3D	AR-Glass	21.2 × 22.5	1000–1700	623	10
2D	AR-Glass	16.9 × 18.1	1000–1700	621.5	/

## Data Availability

No new data were created or analyzed in this study. Data sharing is not applicable to this article.
